# #TreatmentResistantDepression: A qualitative content analysis of Tweets about difficult‐to‐treat depression

**DOI:** 10.1111/hex.13807

**Published:** 2023-06-23

**Authors:** Amelia Talbot, Tori Ford, Sara Ryan, Kamal R. Mahtani, Charlotte Albury

**Affiliations:** ^1^ Nuffield Department of Primary Health Care Sciences, Radcliffe Observatory Quarter University of Oxford Oxford UK; ^2^ Department of Social Care and Social Work Manchester Metropolitan University Manchester UK

**Keywords:** antidepressants, community, depression, qualitative research, treatment resistance, Twitter

## Abstract

**Introduction:**

Treatment‐resistant depression (TRD) is depression unresponsive to antidepressants and affects 55% of British primary care users with depression. Current evidence is from secondary care, but long referral times mean general practitioners (GPs) manage TRD. Studies show that people with depression use Twitter to form community and document symptoms. However, Twitter remains a largely unexplored space of documented patient experience. Twitter data could provide valuable insights into learning about primary care experiences of TRD. In this study, we explored Twitter comments and conversations about TRD and produced patient‐driven recommendations.

**Methods:**

Tweets from UK‐based users were collected manually and using a browser extension in June 2021. Conventional content analysis was used to provide an overview of the Tweets, followed by interpretation to understand why Twitter may be important to people with TRD.

**Results:**

A total of 415 Tweets were organised into five clusters: self‐diagnosis, symptoms, support, small wins and condition experts. These Tweets were interpreted as showing Twitter as a community for people with TRD. People had a collective sense of illness identity and were united in their experiences of TRD. However, users in the community also highlighted the absence of effective GP care, leading users to position themselves as condition experts. Users shared advice from a place of lived experience with the community but also shared potentially harmful information, including recommendations about nonevidence‐based medications.

**Conclusions:**

Findings illuminate the benefits of the TRD Twitter community and also highlight that the perception of a lack of knowledge and support from GPs may lead community members to advise nonevidenced‐based medications.

**Patient and Public Contribution:**

This study was led by a person with lived experience of TRD and bipolar. Two public contributors with mental health conditions gave feedback on our study protocol and results.

## 1 INTRODUCTION

A recent study showed that at least half of adult Internet users find and share health information on social media.^1^ This information offers insight into how people understand and conceptualise their illness and communicate with others about their experiences.^2^ Social media enables access to information that may not be easily collected with other methods.^3^ For example, people with mental health conditions may not want to participate in interviews or focus groups because it can surface traumatic experiences.^4^ However, people often do share their mental health publicly on social media.^1^

Twitter is one of the most popular social media sites, with a global reach of ∼19.05 million users as of October 2021.^5^ Twitter is a microblogging social media site where users can send 280‐character messages or ‘Tweets’. Twitter profiles are public, except for users who have set privacy settings. This is compared to Facebook, where users tend to have private profiles and mostly interact with friends and family.^6^

Evidence shows that mental health can improve by talking to friends and family.^7^ Therefore, it is reasonable to assume that Facebook would be an appropriate social media for mental health discussions. In qualitative studies, however, people have said they prefer Twitter. For example, in Berry et al.'s study,^8^ participants perceived people on Twitter to be more authentic and less judgemental about mental health. This suggests that there would be more discussion on Twitter about treatment‐resistant depression (TRD) than on other social media.

Studies have focused on the potential associations between depression and increased Twitter use. For example, a recent quantitative study found that excessive Twitter use was associated with depression among university students.^9^ A regression analysis also found associations between mental health crises and the consumption of Twitter mental health content.^10^ However, in qualitative studies, people with mental health conditions have reported using Twitter to form a community, document symptoms, and safely express themselves.^8^ These findings illustrate our limited understanding of the relationship between Twitter and mental health. Twitter could be a valuable resource for learning about this relationship. Users often Tweet complex, in‐the‐moment experiences they might not recall in interviews.^11^

Mental health Twitter studies have focused on hashtags like #MyDepressionLooksLike,^12^ #WhyWeTweetMH,^8^ #DearMentalHealthProfessionals^13^ and #Schizophrenia.^14^ In these studies, people Tweeted about medication, crisis planning and service provision. However, it is unclear if these Tweets transfer to TRD. TRD is depression that does not respond to antidepressants^15^ and is consistently linked to high economic burdens due to the increased use of health services, increased cost of care, poor quality of life and loss of productivity.^16^, ^17^, ^18^ Approximately 55% of UK primary care users have TRD.^19^ People with TRD should be referred to secondary care, but long wait times prevent this from happening.^20^, ^21^

There is limited guidance for general practitioners (GPs) on managing TRD,^21^ and people with TRD have described dissatisfaction with GP care.^22^ Therefore, it might be important to investigate how to improve health experiences and how GPs can be supported in caring for these people. Much research on TRD has focused on secondary care, despite the long wait times mentioned above.^22^ This may mean that a large cohort of people with TRD is not reflected in the research. This study addresses this research and service gap. Tweets could provide feedback about how people experience primary care mental health services.

Twitter is increasingly used in clinical training and continuing professional development. For example, a qualitative study found that GPs sometimes read #TipsForNewDocs to improve their knowledge of the patient experience.^23^ A systematic review also found that health‐related Tweets were described as useful when incorporated into undergraduate education and continuing professional development.^24^

This study presents a qualitative content analysis^25^ of Tweets about TRD. We aimed to identify *what comments and conversations are posted on Twitter about TRD*. Understanding the content people with TRD share on Twitter could support GPs and researchers in understanding people's experiences.

## 2 METHODS

A patient‐led methodology^26^ was used, meaning it was led by A. T., who has lived experience with TRD and bipolar.

### 2.1 Data collection

A. T. (a female, young adult, PhD student and qualitative researcher) developed a search strategy using synonyms for TRD found in Brown et al.'s systematic review.^27^ Synonyms included: ‘chronic’, ‘complex’, ‘difficult to treat’, ‘enduring’, ‘life‐long’, ‘long‐term’, ‘major’, ‘multiple episodes’, ‘persistent’, ‘recurrent’, ‘relapse’ and ‘treatment‐resistant’. The strategy was discussed via email with two public and patient contributors (PPI) with mental health conditions. PPI additionally suggested including terms with and without hashtags.

A. T. entered search terms into NCapture,^28^ a web browser extension that gathers web content for direct importation into NVivo.^29^ NCapture^28^ was not gathering sufficient Tweets, collected mostly research and retweets and could not capture replies. Therefore, A. T. stopped using NCapture and supplemented data with a manual method of collecting Tweets, which she called ‘Tweet‐Chasing’. This involved looking at the original users' feeds (identified through NCapture) and then collecting relevant data from (a) their interactions with others, (b) those other users' feeds and (c) those other users' interactions. A. T. uploaded relevant Tweets identified through Tweet‐Chasing into NVivo.^29^ A. T. collected replies to Tweets where the user had reported in the Tweet or their biography (bio) that they had TRD.

A. T. collected Tweets using NCapture^28^ on 30 June 2021, with 6431 Tweets collected. A total of 6384 of these Tweets were excluded (see next section for inclusion criteria and results for reasons for exclusion), leaving 47 eligible for analysis. A. T. collected an additional 368 Tweets via Tweet‐Chasing between 20 July and 2 August 2021. The final sample was 415 Tweets, comparable to the amount used in similar studies.^8^, ^30^, ^31^, ^32^

A. T. followed a pragmatic approach to sampling,^33^ stopping during analysis when she interpreted there to be adequate data to support findings within the practicalities of the research. A. T. did not use saturation because she believes researcher subjectivity means that new interpretations can always be made.^33^

### 2.2 Exclusion criteria

A. T. excluded Tweets manually against the following criteria:

1. Advertisements (including for research).
2. One word or hashtags. It would be difficult to extract meaning from these Tweets.
3. Private profile.
4. Published outside of the United Kingdom (and non‐English language). We focused on the United Kingdom because of the different cultural contexts pertaining to healthcare (e.g., private healthcare in America). Users can report locations in bios; an approximate location is reported on NCapture.^28^
5. User is a health, governmental or charitable organisation.
6. User is suspected to be <18 (e.g., mentions Child and Adolescent Mental Health Services).

Search terms were excluded during Tweet‐Chasing, where users reported living with TRD in their bio or prior Tweet. This ensured that contextually relevant Tweets were collected. Demographics were not collected as they were not reported in bios. Likes and retweets were ignored as our question was not about Tweet popularity. Replies were collected as part of Tweet‐Chasing if the user had reported living with TRD in their bio or previous Tweet. There was no date limit on Tweets. We made this decision because we were likely to find Tweets from the past few years, as Twitter limits how far you can go into the archives. The full search strategy can be found in the registered report.^34^

### 2.3 Analysis

A. T. coded the Tweets qualitatively using Hsieh and Shannon's^25^ conventional content analysis. This inductive method involved developing ‘descriptive clusters’ to provide condensed descriptions of the contents of Tweets.^25^ The process involved: (i) reading the Tweets and noting down initial ideas/codes; (ii) coding each Tweet in NVivo^29^ descriptively, with some Tweets coded twice if they contained multiple meanings; (iii) checking that each Tweet was coded appropriately; (iv) merging similar codes into clusters; (v) calculating how many Tweets were coded for each cluster and (vi) writing up these clusters. Consistent with conventional content analysis,^25^ we did not contextualise the clusters with existing literature until the discussion.

A. T. interpreted the descriptive clusters using the One Sheet of Paper (OSOP) method.^35^ The OSOP^35^ involved mind mapping all codes and their relationships on OSOP to identify the line of argument across clusters. Deviant cases were included in the OSOP. A. T. created one latent (interpretative) cluster from the OSOP method that described why Twitter might be important to people with TRD. Coding was developed iteratively with feedback from all authors and PPI. All authors agreed with the final clusters.

Our ontological approach was grounded in critical realism,^33^ following a view that there is one social reality, but subjectivity limits our understanding of it.^36^ The team followed Ahuvia's^37^ conception of content analysis, which states that content analysis is interpretive, not simply descriptive, as the researcher's subjectivity makes pure description impossible. This means that our resulting descriptive clusters are not ‘counts of content’, but ‘counts of our interpretations’ of the Tweets.^37^

### 2.4 Ethical considerations

The University of Oxford approved this study (reference: R76585/RE001). The British Psychological Society^38^ considers Twitter part of the public domain, where users can expect to be used in research without consent. We decided that because Tweets are public, does not mean users give up their privacy. We developed a careful plan with PPI to safeguard users. The plan included removing identifiers and paraphrasing Tweets in a way that retained their original meaning. A. T. re‐entered paraphrased Tweets into Twitter to ensure they could not be traced back to users. This approach is congruent with similar studies.^8^, ^30^, ^31^

The team also planned how to safeguard any users expressing suicidal ideation. We reported these to Twitter so that users could receive crisis resources. Evidence shows that 14% of suicide‐related Tweets require intervention.^39^

## 3 RESULTS

### 3.1 Description of tweets

**Figure 1:**
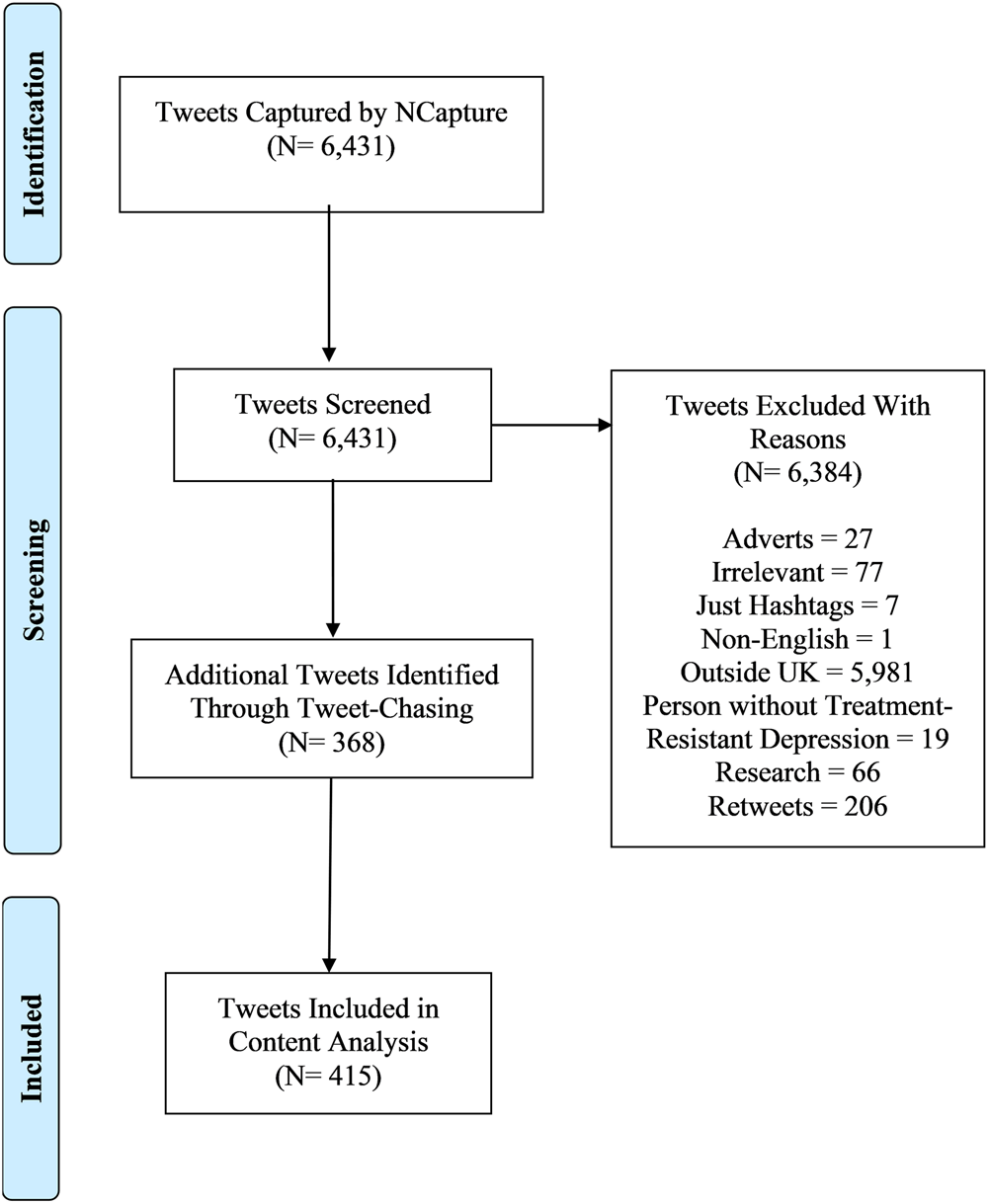
Data collection process.

Of the 6431 Tweets found by NCapture, we excluded 6384. The reasons for exclusion are shown in Figure 1. We Tweet‐Chased 368 Tweets, totalling 415.

Tweets were posted between December 2017 and August 2021 by 152 users. Tweets were posted between December 2017 and August 2021 by 152 users. The longest Tweet was 240 characters, and the shortest was 20 characters (range = 220, mean = 182). Frequent words included depression (*N* = 389, 94%), treatment (*N* = 118, 28%) and resistant (*N* = 111, 27%). The most common synonyms for treatment resistance (*N* = 115, 27%) were life‐long (*N* = 52, 13%) and chronic (*N* = 46, 11%).

### 3.2 Descriptive clusters

We developed five clusters to describe the Tweets: (1) self‐diagnosis: I think I have TRD; (2) symptoms: I experience helplessness, exhaustion and suicide ideation; (3) peer support: I offer advice to others with TRD; (4) small wins: This is what I can do despite my TRD and (5) condition expert: I know my TRD best. Clusters are introduced with a number and percentage to demonstrate how many Tweets were coded for each cluster. Consistent with other conventional content analyses, we do not use numbers/percentages elsewhere.^8^ The number of users who Tweet something does not show how insightful or important it is for answering our research question. Every Tweet in this section comes from a different user.

### 3.3 Cluster One: Self‐diagnosis

We interpreted 26 (6%) of Tweets to relate to users self‐diagnosing with TRD. Some users said that even if they had a ‘well‐documented’ history of not responding to antidepressants, their doctors had not considered treatment resistance:

My doctor did not diagnose me [with TRD]. He was reviewing my antidepressant medication, and I was recounting my depression symptoms to him, with many years of well‐documented history of depression treatment

…Nobody has ever diagnosed me, but I understand my depression as persistent and treatment‐resistant

Some users Tweeted that they were diagnosed with TRD. However, these users said that they knew they had TRD before being diagnosed:

Have just been diagnosed with TRD, which I already knew

A few users may have self‐diagnosed TRD because of Twitter interactions:

Someone on Twitter suggested that my condition could be TRD. I got help and learned a lot about myself…

Users' reactions to self‐diagnosis varied. Two users described the thought as ‘scary’ and ‘frightening’. Four users said it was a relief to have an explanation for why antidepressants were not working. A self‐diagnosis helped these users reconcile with their symptoms and understand that their lack of response was not their fault:

TRD sucks, but when I learned about it, I felt relieved. Because when you do not respond to treatment, you start to think it is your fault for not trying hard enough

One user said it saved their lives to have an explanation for why antidepressants did not work:

It saved my life when I self‐diagnosed myself with TRD. Knowing I may never respond to treatment, you may think it would make me feel worse, but actually, it was a relief

### 3.4 Cluster Two: Symptoms

We interpreted 148 (36%) Tweets as describing symptoms of TRD. Symptoms included exhaustion (*n* = 19), helplessness (*n* = 37), sadness (*n* = 31), self‐harm (*n* = 40) and suicide ideation (*n* = 21). We describe each of these below. Many users described feeling helpless and, like their depression, would never improve:

Because of my depression, I feel like things will never change; I have lost hope about the situation; it's hard to look after myself

Some of these users mourned for a life without depression. The following user said that it hurt to imagine what their life would have been like had they not had depression and their other conditions:

I have fibro issues, chronic pain, and TRD. I think about the loss of what could have been, the little and big things… it hurts

Many users Tweeted about self‐harm and suicide ideation. Several users described their self‐harm methods, and others expressed their identity as a ‘suicide survivor’:

I am a suicide survivor. TRD is painful

Many users described feeling exhausted. Some of them wished their depression would end:

I am absolutely exhausted from my TRD; I wish it would stop; I wish it were not like this

A couple of users wished that people without depression would understand how much energy is required to do simple things (e.g., making a bed, brushing their teeth):

I have TRD. I wish normies [people without depression] would understand how exhausting it is and how much it takes to do simple things

### 3.5 Cluster Three: Peer support

We coded 81 Tweets (20%) as peer support. Most users shared how they managed their own TRD when providing peer support. Self‐management included practising resilience and not watching potentially triggering movies:

TRD is dreadful, especially when you have those deep patches. It nearly claimed me too, but it will not and will not claim you either

Many users said that they had found a way to manage their TRD and told readers with the condition to ‘not give up’:

I have been living with TRD and anxiety since 2004. I finally feel stable with the right medication. I am getting better, do not give up

Direct messages were interpreted as an additional channel of support:

There are support groups that have helped me. Not sure where you are located, but here is the group I use [link]. DM me if you need help

### 3.6 Cluster Four: Small wins

We coded 62 (15%) Tweets as ‘small wins’—accomplishments that might seem easy, but they are significant when considered in the context of cluster Two (symptoms). Indeed, users appeared to experience severe, debilitating symptoms. Yet, they were still able to achieve ‘small wins’ like getting out of bed, going outside, or doing something that made them anxious:

I have TRD, and it gets worse without human contact. But I am doing well, considering. I have a lot of hobbies, and I tidied most of my house today

Many users appeared to feel proud of their progress with their TRD. They described working for a long‐time to become well‐managed. This success was illustrated with words like ‘proud’ and ‘I did it’:

I have had TRD for years. I have worked very hard on my mental health. Proud to say my mental health is the best it has been in some time

Users decided to complement their antidepressants with self‐reflection, counselling or mindfulness. It was unclear whether health professionals offered these alternatives:

I got my TRD well‐managed with antidepressants, counselling, lifestyle change, and mindfulness

Another small win was accepting depression as a potentially long‐term condition. One user said their support network helped them achieve acceptance:

I have had TRD for most of my life. In the last couple of years, I have come to a place of acceptance and peace that I did not think possible. I have an excellent support network who have helped me

### 3.7 Cluster Five: Condition expert

We perceived 40 users (10%) as viewing themselves as experts on self‐diagnosing and coping with TRD. We interpreted the lack of perceived quality GP care as why users established themselves as condition experts. For example, referring back to Cluster One (self‐diagnosis), users mentioned wanting an explanation for why antidepressants did not work. When GPs (and other health professionals) did not explain and continued with recurrent antidepressant prescribing, users took on the role of diagnosis and self‐diagnosed TRD:

I was told by my therapist that my doctors finally recognise TRD, even though this is how I have understood my depression for some time

The condition expert role also manifested in regards to treatment. For example, some users said they decided to stop taking antidepressants after trying ‘every single antidepressant the NHS can give’. One user perceived themselves to no longer be treatment‐resistant after stopping antidepressants. It was unclear whether this user spoke to their GP about this decision, and we do not know if their recovery was linked to stopping antidepressants:

I decided to stop taking my antidepressants and am cured of my TRD

This establishment of being a conditioning expert resulted in many users sharing their expertise with other users. Some of these Tweets relate to Cluster Three (peer support), where users shared what they did to manage their TRD. A couple of users, for example, shared how their symptoms reduced with St John's Wort (herbal medication). Although there are risks to self‐prescribing,^40^, ^41^ it was evident that a lack of support from GPs led users to take these medications and give and receive medical advice:

How about St John's Wort? I tried it, and it really has helped my depression. I found it all by myself. Not sure my GP knows it exists…

Indeed, many users described not having any choice but to become condition experts and manage their treatment on their own when their GPs offered no support beyond antidepressants:

I have come to realise that many GPs are dumb; you really have to advocate for your own body and needs

There were benefits from users sharing their experiential expertise, including reflections on TRD improving over time (Cluster Three). However, most users described wanting to combine their experiential expertise with the clinical expertise of a GP. These users described rejecting the advice from GPs who told them to ‘get over’ their depression and searching for GPs who understood appropriate treatment pathways for TRD:

I changed my GP several times. They kept saying I should ‘get over it’ and ‘pull myself together’. I cannot be treated by someone who doesn't understand the situation

### 3.8 Latent cluster

Our interpretation of descriptive clusters showed that Twitter could be a community for people with TRD. Users could connect, form new friendships, ask and receive advice and share experiences with GPs and other health professionals. This interpretation is captured in the latent cluster ‘supportive community’. The majority of Tweets in this section contain ‘@username’. The @ sign indicates a response to a user/Tweet. We interpreted these @s as a sign of mutuality and engagement with the community.

### 3.9 Latent cluster: Supportive community

**Figure 2:**
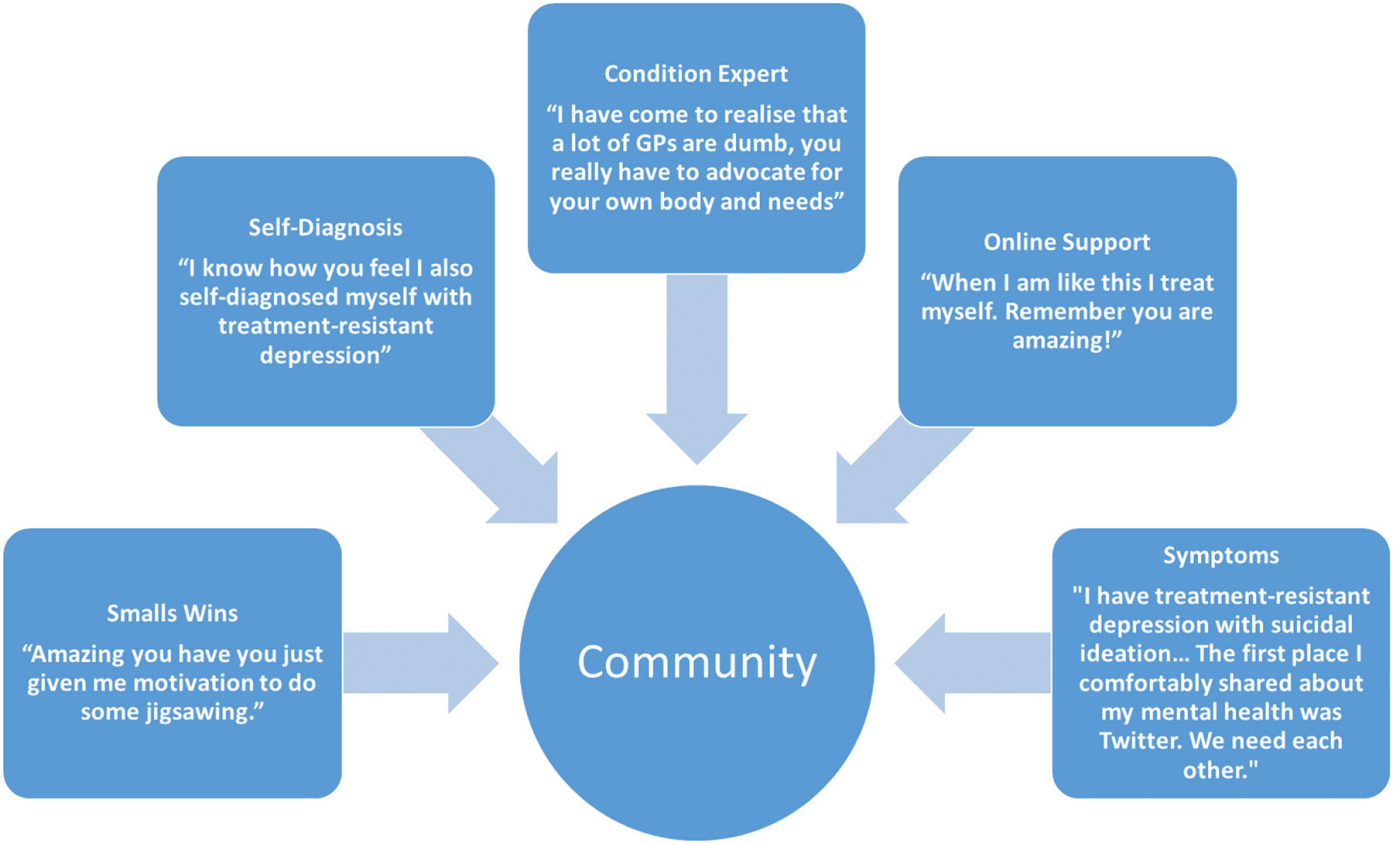
Community cluster.

Analysis of the descriptive clusters highlighted Twitter as a positive and supportive community for users with TRD (Figure 2). There are many online communities where users promote harmful content and misinformation.^42^, ^43^, ^44^, ^45^ However, we interpreted the users in this community as friendly, empathetic, and helpful. These community users are described as valuable in the absence of GP care, saying, for example, ‘This community is incredible’ and ‘I love this community’. Some evidence was latent within the text. For example, several users used the word ‘we’, which we interpreted as signifying reciprocity with other users. Several Tweeted, ‘We need each other’, ‘We've been there’, and ‘We're here for you’. Users foregrounded how Twitter enabled them to connect with a wider community of people with TRD and access support:

@username. Thank you for Tweeting this when you are so feeling low. If you want to talk, just DM me. I am feeling very depressed too. We are here to listen

@username. I also have TRD. I have tried every antidepressant. I find it hard to talk about, but I just want to send you love

Twitter was described as a place for users to share their experiences, ask for help, gain and offer support. Indeed, many users described using Twitter to connect with like‐minded people and access support they might not have had access to elsewhere. Several of these users used words like ‘thank you’, which we interpreted as users being grateful for the supportive community that Twitter can provide:

Thank you to everyone who responded to my previous Tweet about my depression. I was nervous to Tweet about something so personal, but Twitter is supportive, and this community is incredible. Thank you, it means the world to me

Users described feeling comfortable engaging and receiving support from the Twitter community. They said that Twitter was the only place they felt comfortable talking about their TRD:

I have TRD with suicidal ideation, anxiety, panic disorder, ADHD, and alcoholism. The first place I openly and comfortably shared about my mental health was Twitter. We need each other

Users also appeared to be comfortable sharing their experiences with GPs for TRD on Twitter. For example, one user said their GP refused to refer them for their self‐diagnosed TRD. Among the responses: ‘What?!’, ‘Report that’ and ‘I experienced the same thing!’ This perception of poor quality GP care led to users positioning themselves as condition experts responsible for diagnosing and treating TRD, as discussed in Cluster Five (Condition Expert). Users' role as condition experts seemed to make them comfortable with sharing and receiving advice, including groups, hotlines and crisis resources:

@username. Have you tried the Samaritans? They are great at snapping you out of suicide ideation

@username. When I am like this, I treat myself. Remember, you are amazing

These self‐management tools are evidence‐based and recommended by the UK National Institute for Health Care Excellence guidance.^46^ However, users also sometimes shared and encouraged other users in the community to take potentially unsafe, nonevidenced‐based medications (e.g., St Johns Wort; Cluster Two), abruptly stop taking antidepressants (Cluster Five) and incorrectly diagnose themselves with TRD (Cluster One). This means that while users in the community were kind and supportive, some Tweets contained harmful content and could expose users to potentially dangerous information.

### 3.10 Reflexive statement

The Tweets in our analysis come from users with TRD. A. T., who is part of the Twitter TRD community, suggest that users could also be exposed to harmful information from noncommunity members. Indeed, A. T. has received Tweets about nonevidence‐based pseudoscientific diets and medications in response to some of her Tweets about TRD. A. T. usually ignores these Tweets and blocks the user, but it is unlikely that every user with TRD will do the same. Indeed, you may be willing to try anything when you live a severe, life‐limiting condition like TRD (Cluster Two). A. T.'s experiences are supported by an interview study by Morris et al.,^47^ who found that social networks influence perceived support needs and attitudes to self‐management.

## 4 CONCLUSIONS

Our qualitative study shows that people with TRD experience the Twitter community as positive and supportive. Twitter can help people connect with others and access support anytime and when they do not receive care from GPs. These findings are consistent with studies on mental health generally^8^ and non‐TRD.^12^ People asserted themselves as ‘condition experts’ expressing dissatisfaction with care from their GPs. In asserting this expert role, members of the TRD community suggested medications to each other that were untested, unsafe and nonevidenced based. This included information regarding St John's Wort, which has mixed evidence for its effectiveness and safety for major depression.^48^

This role of the condition expert seemed to result from perceived low quality and lack of GP care. Indeed, most users described taking responsibility for diagnosis and treatment when GPs ignored their nonresponse to antidepressants. This absence of GP care resulted in some users self‐diagnosing and encouraging others to self‐diagnose TRD (Cluster One). Giles and Newbold^49^ found that self‐diagnosis after interacting with social media is common among people with mental health conditions. Self‐diagnosis was described as helping some users reconcile with their nonresponse—supporting Lewis's^50^ interview study on self‐diagnosed autism. However, self‐diagnosis can also be dangerous because of the associations between self‐diagnosis and medical misinformation, health anxiety and misdiagnoses.^51^ This self‐diagnosis, if accepted by clinicians, could escalate into more serious, incorrect and intensive treatments like ketamine which can be costly to the NHS and cause side effects like headaches, fatigue and increased suicide ideation.^52^

The lack of GP support beyond recurrent antidepressants might result from the term TRD not being commonly used in primary care and may be thought to describe those in secondary care.^15^ Yet, as the authors have said in a previous paper^22^ and have been told by others working on TRD,^15^, ^53^ many people in primary care continue having inadequate responses to antidepressants and describe the feeling that the term usefully describes their symptoms. Without GP care, people may turn to the Twitter community for support which could expose them to both supportive and harmful information. GP training and continuing professional development may support GP awareness of TRD and help them become familiar with appropriate treatment pathways beyond recurrent antidepressants. More active management of people with TRD may also improve outcomes for this group.^15^

We interpret Twitter as both advantageous and disadvantageous to people with TRD. Users can find support and community anytime while exposed to a potentially inaccurate self‐diagnosis and encouragement to withdraw antidepressants and try nonevidence‐based medications. These interpretations align with Susi et al.'s^54^ systematic reviews that found viewing self‐harm images online can have harmful (encouragement to self‐harm) and protective effects (access to peer support). Susi et al.'s^54^ study and ours show the importance of GPs assessing an individual's access to images and information related to TRD, self‐harm and suicide.

Users clearly valued the community aspect of Twitter, reflecting people's experiences with offline mental health communities.^55^ These communities value and allow people to share their experiential expertise, but the information shared is usually moderated to address potentially distressing and nonevidence‐based information.^55^ GPs may wish to encourage people to join local support groups to be involved with a moderated, supportive community.

Our study was exploratory, so we did not use theory. However, our results may show that an advantage of using Twitter is that users with TRD can increase their social capital. Social capital posits personal relationships as resources that increase human functioning.^56^ Social capital can be lower among people with depression, resulting in fewer social connections and fewer opportunities for support.^57^ Our study showed how users could form new social connections with people with TRD on Twitter. Many users described their relationships with community members as supportive and reassuring. This kind of support, some users said, was not available elsewhere. Our interpretations are supported by a quantitative content analysis of online depression forums by Pan et al.^58^ Again, offline community groups may contribute to the growth of social capital in a safe and moderated environment.^59^

### 4.1 Strengths and limitations

We have contributed to the small qualitative literature base on TRD and primary care.^22^ NCapture did not identify relevant tweets, so we developed the ‘Tweet‐Chasing’ method. We perceive this method as useful for future Internet‐mediated research. We used conventional and latent content analysis. This method allowed us to summarise and develop depth and meaning from the Tweets rather than operating at just the surface level. This study was led by A. T., a person with TRD and bipolar who has experienced Tweeting about mental health. A. T. perceived her experiences as a useful tool for interpretation.

On limitations, Twitter has a 280‐character limit, so Tweets lack context and may have been misinterpreted. Tweets were posted between December 2017 and August 2021, but some relevant Tweets will have been missed. Researchers have suggested that negative experiences can be more heavily weighted in Twitter studies.^30^ The cluster ‘small wins’ shows that users Tweet positively about their TRD, so this limitation does not necessarily apply. There may have been recall and social desirability bias. Tweets were from active users, so we cannot learn from or understand the reasons why some people may have left Twitter. It is unknown whether users' self‐diagnoses of TRD align with clinical definitions. This research was conducted before Elon Musk bought Twitter (October 2022). It is unknown whether Musk's purchase of Twitter has affected the community reported here. However, Twitter's monthly active users are growing.^60^

### 4.2 Conclusions

Our qualitative study of Tweets about TRD found that Twitter provides a positive, supportive community where people with similar illness experiences ask for and receive advice. Twitter can help people reconcile with symptoms and find community, supporting self‐management in the absence of GP care. However, the advice shared was not always underpinned by evidence, and users sometimes recommended nonevidence‐based medications. Our findings illuminate the benefits of the TRD Twitter community and also highlight that the perception of a lack of knowledge and support from GPs may lead community members to try and advise untested medications.

### AUTHOR CONTRIBUTIONS

**Amelia Talbot**: Conceptualisation; data collection; formal analysis; funding acquisition; methodology; project administration; software; original draft; writing; review; editing. **Tori Ford**: External audit; review; editing. **Sara Ryan**: Funding acquisition; methodology; supervision; review; editing. **Kamal R. Mahtani**: Supervision; review; editing. **Charlotte Albury**: Funding acquisition; methodology; supervision; review; editing.

## CONFLICT OF INTEREST STATEMENT

The authors declare no conflict of interest.

## ETHICS STATEMENT

The study was ethically approved by the University of Oxford Medical Sciences Interdivisional Research Ethics Committee (Reference: R76584/RE001).

## Data Availability

Research data are not shared due to using Tweets that could make individuals identifiable.
